# Objective analysis of facial bone fracture CT images using curvature measurement in a surface mesh model

**DOI:** 10.1038/s41598-023-28056-7

**Published:** 2023-02-02

**Authors:** Jeenam Kim, Chaneol Seo, Jung Hwan Yoo, Seung Hoon Choi, Kwang Yeon Ko, Hyung Jin Choi, Ki Hyun Lee, Hyungon Choi, Donghyeok Shin, HyungSeok Kim, Myung Chul Lee

**Affiliations:** 1 Department of Plastic and Reconstructive Surgery, School of Medicine, Konkuk University, Seoul, Korea; 2 Department of Computer Science and Engineering, Konkuk University, 120 Neungdong-Ro, Gwangjin-Gu, Seoul, 05030 Korea

**Keywords:** Biotechnology, Computational biology and bioinformatics, Mathematics and computing

## Abstract

The human facial skeleton consists of multiple segments and causes difficulty during analytic processes. We developed image analysis software to quantify the amount of injury and validate the smooth curvature of the surface after facial bone reduction surgery. Three-dimensional computed tomography images of facial bone were obtained from 40 patients who had undergone open reduction surgery to treat unilateral zygomaticomaxillary fractures. Analytic software was developed based on the discrete curvature of a triangular mesh model. The discrete curvature values were compared before and after surgery using two regions of interest. For the inferior orbital rim, the weighted average of curvature changed from 0.543 ± 0.034 to 0.458 ± 0.042. For the anterior maxilla, the weighted average of curvature changed from 0.596 ± 0.02 to 0.481 ± 0.031, showing a significant decrement (*P* < 0.05). The curvature was further compared with the unaffected side using the Bray–Curtis similarity index (BCSI). The BCSI of the inferior orbital rim changed from 0.802 ± 0.041 to 0.904 ± 0.015, and that for the anterior maxilla changed from 0.797 ± 0.029 to 0.84 ± 0.025, demonstrating increased similarity (*P* < 0.05). In computational biology, adequate analytic software is crucial. The newly developed software demonstrated significant differentiation between pre- and postoperative curvature values. Modification of formulas and software will lead to further advancements.

## Introduction

The facial skeleton consists of multiple convoluted bony segments, and injury can be diagnosed using various imaging methods^1^. In contrast to the analysis of other long bones, the structural characteristics of facial bone increase the difficulty of the diagnostic process^2^. In case of long bone injuries, such as hand fractures, biomechanical studies can be used to analyze the recovery of the fracture site^3^. However, facial bones are difficult to evaluate using biomechanical studies such as applying impact or weight loading. Thus, to visualize facial fractures, computed tomography (CT) studies are performed before and after surgery^4^. These studies provide useful information to determine whether the fracture site has been adequately reduced. It is necessary to confirm that the curve of the bone-to-bone contact area exhibits a smooth union.

Biologic imaging techniques such as CT scans have been utilized in static as well as dynamic modalities. Structural disfigurements are visualized when a natural alignment is broken. On the other hand, recovery of disarranged anatomical structures can be confirmed efficiently in postoperative morphometric evaluation^5^. Cell deformation or movement is another issue to be considered, and epithelial stiffness and deforming force provide information. The spontaneous curvature of cellular mesh structures can be a solution to figure out the dynamic change^6^. Furthermore, facial bone components possess a viscoelastic characteristic. The unexpected alteration requires exquisite images to validate an adequate correction^7^.

Therefore, image analysis software was developed to determine whether the facial bone surface achieves a smooth curve after corrective surgery. The discrete curvature of a triangular mesh model was utilized in the assessment process^8–10^. Various polyhedral mesh models have been introduced to evaluate a convoluted surface preserving innate topology^11^. The triangular mesh model of our software was based on a pyramid consisted of multiple triangles. When the apex of pyramid is converted from sharp to blunt morphology, apical angles alter subsequently. Trigonometric functions using the angles provide adequate information for objective analysis.

The program was applied to a specific region with either a smooth curve or substantial bends of the facial bone surface. The discrete curvature values were calculated using pre- and postoperative images. Before surgery, a tendency of substantially bent areas in the facial bone could exist. After surgery, smooth curves should be exhibited if appropriate reduction has been achieved. Here, we describe the development of the analysis software and present the outcome measures in this report.

## Results

**Figure 1:**
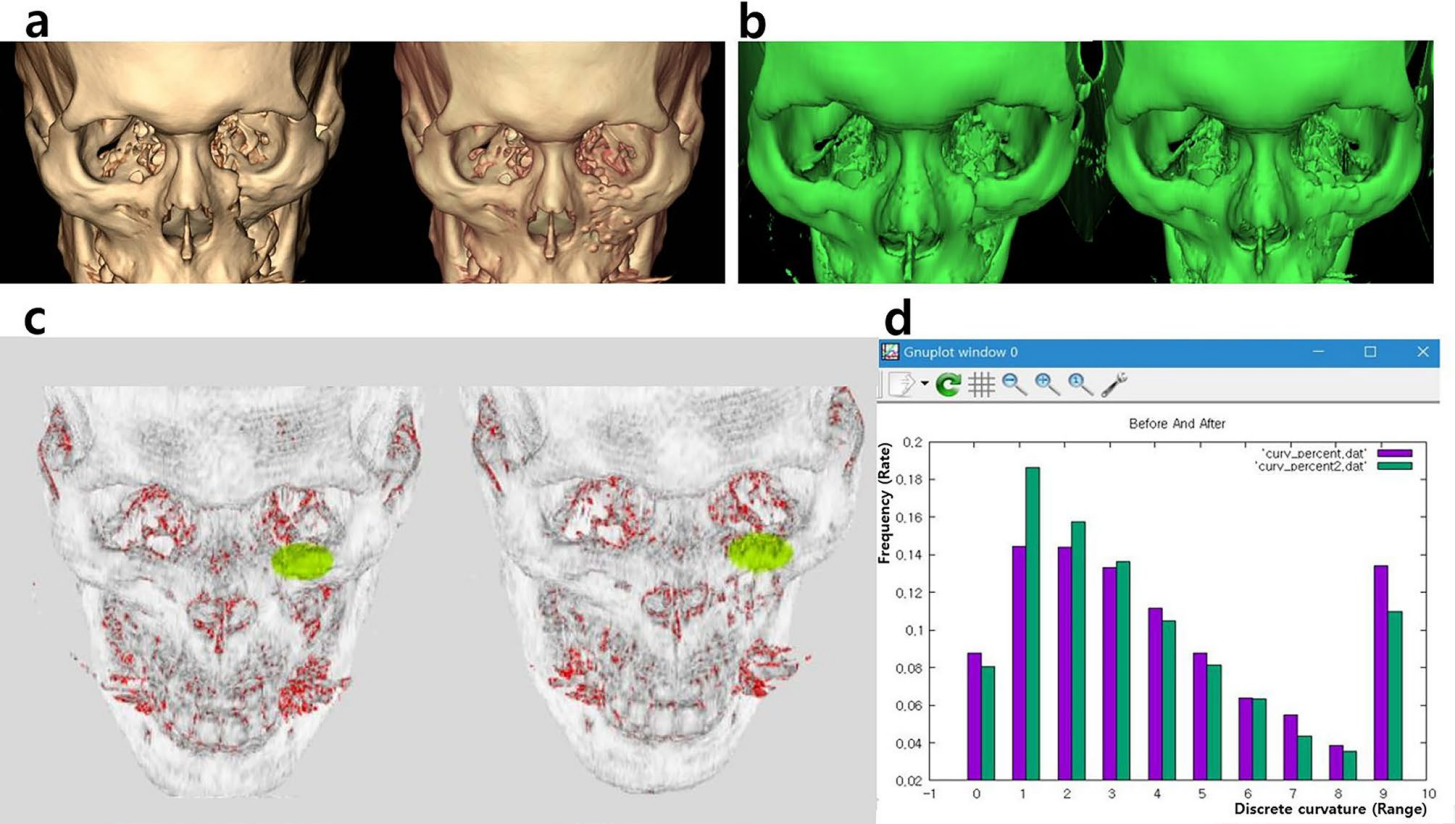
Analysis of the inferior orbital rim. (**a**) Fractured left inferior orbital rim was exhibited preoperatively and displaced segment was reduced postoperatively. (**b**) The initial medical images in a DICOM file format were converted into OBJ files. (**c**) During image processing, a specific area (the part reduced during surgery) was selected and the vertices included in the selected area were displayed in fluorescent green. (**d**) The discrete curvature mainly showed large values preoperatively (purple bars), and the range shifted toward smaller values postoperatively (green bars). The range of large values-high curvature (from 4 to 9) was more dominant preoperatively, and the range of small values-low curvature (from 1 to 3) postoperatively. The weighted average of curvature changed from 0.52 to 0.46.

**Figure 2:**
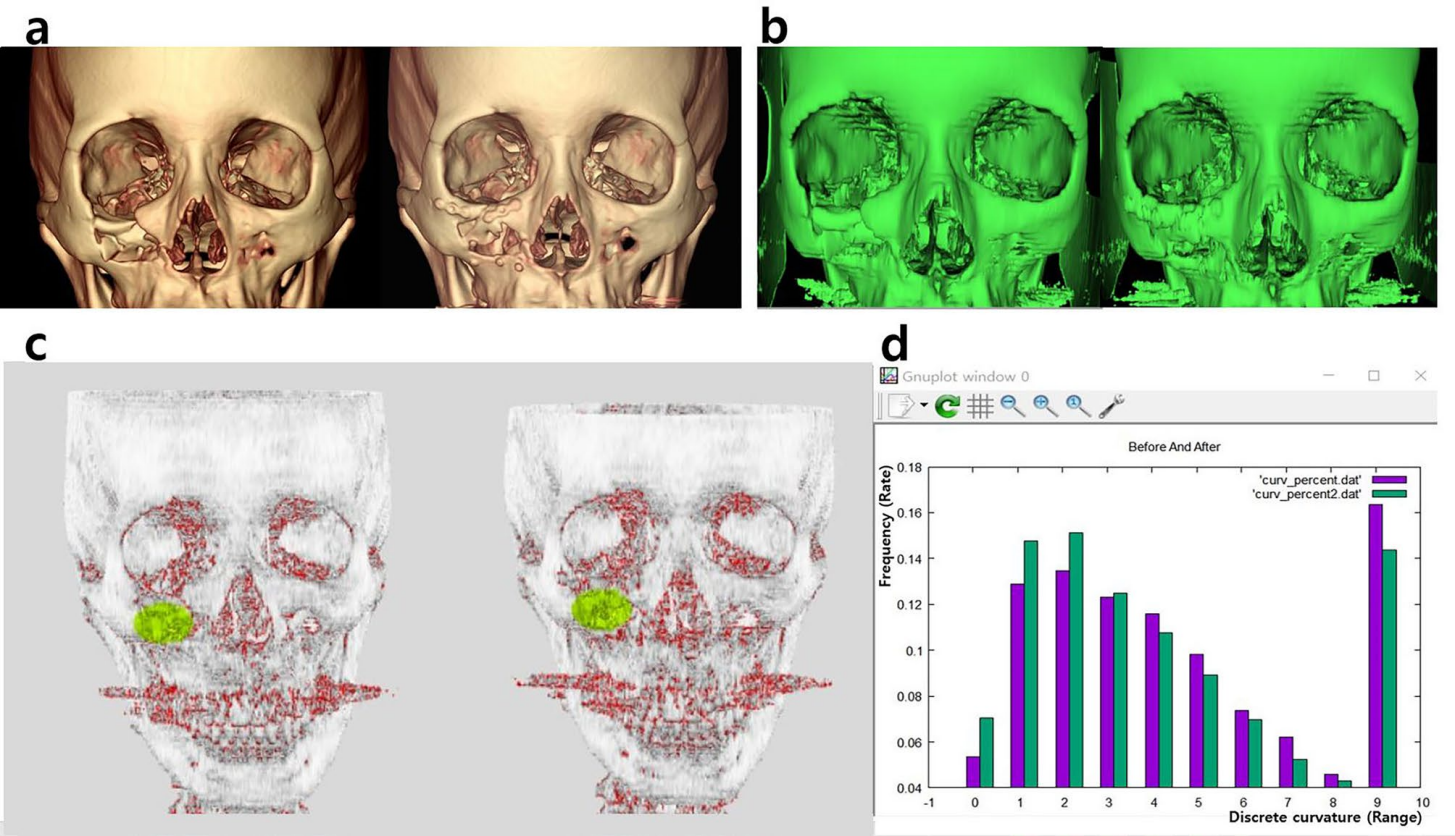
Analysis of the anterior maxilla. (**a**) Fractured right anterior maxilla was noted preoperatively and the segment was reduced postoperatively. (**b**) The initial DICOM imaged were converted in to OBJ files. (**c**) The subject areas were selected and displayed in fluorescent green. (**d**) The discrete curvature mainly showed large values preoperatively (purple bars), and the range shifted toward smaller values postoperatively (green bars). The range of large values-high curvature (from 4 to 9) was more dominant preoperatively, and the range of small values-low curvature (from 0 to 3) postoperatively. The weighted average of curvature changed from 0.58 to 0.47.

The discrete curvature values obtained before and after surgery using the software were compared. The weighted average of curvature of the inferior orbital rim before surgery was 0.543 ± 0.034, and that after surgery was 0.458 ± 0.042 (*P* < 0.05). The weighted average of curvature of the anterior maxilla before surgery was 0.596 ± 0.02, and the value after surgery was 0.481 ± 0.031 (*P* < 0.05). A comparison of the weighted average values before and after surgery confirmed that the values decreased for both the inferior orbital rim and anterior maxilla. Additionally, the graphs demonstrated that the curvature was steep before the operation and became smoother after surgery (Figs. 1 and 2).

The outcomes verified that the image analysis software could detect a smooth surface of a curve after reduction surgery. The analysis software can be applied to regions with smooth curves or sharply curved bends on the facial bone surface.

The curvature value was further compared with the curvature of the unaffected side. Because our study included patients with unilateral zygomaticomaxillary fractures, a comparative analysis could be performed between the fractured and unaffected sides. The Bray–Curtis similarity index (BCSI) was used to calculate the inter-unit similarity between the pre- and postoperative images. The BCSI has been utilized in analyses comparing compositions between ecological communities and for validation of ingredients in different formulas^12,13^.

1 $$BC_{ij} = \frac{{2C_{ij} }}{{S_{i} + S_{j} }}$$

The BCSI outcome values range from 0 to 1, and larger values represent more similar components between groups. Calculating the BCSI using Eq. (1), *C*_*ij*_ is the sum of the lesser values, and *S*_*i*_ and *S*_*j*_ are the total numbers of collective data counted at both sites.

**Figure 3:**
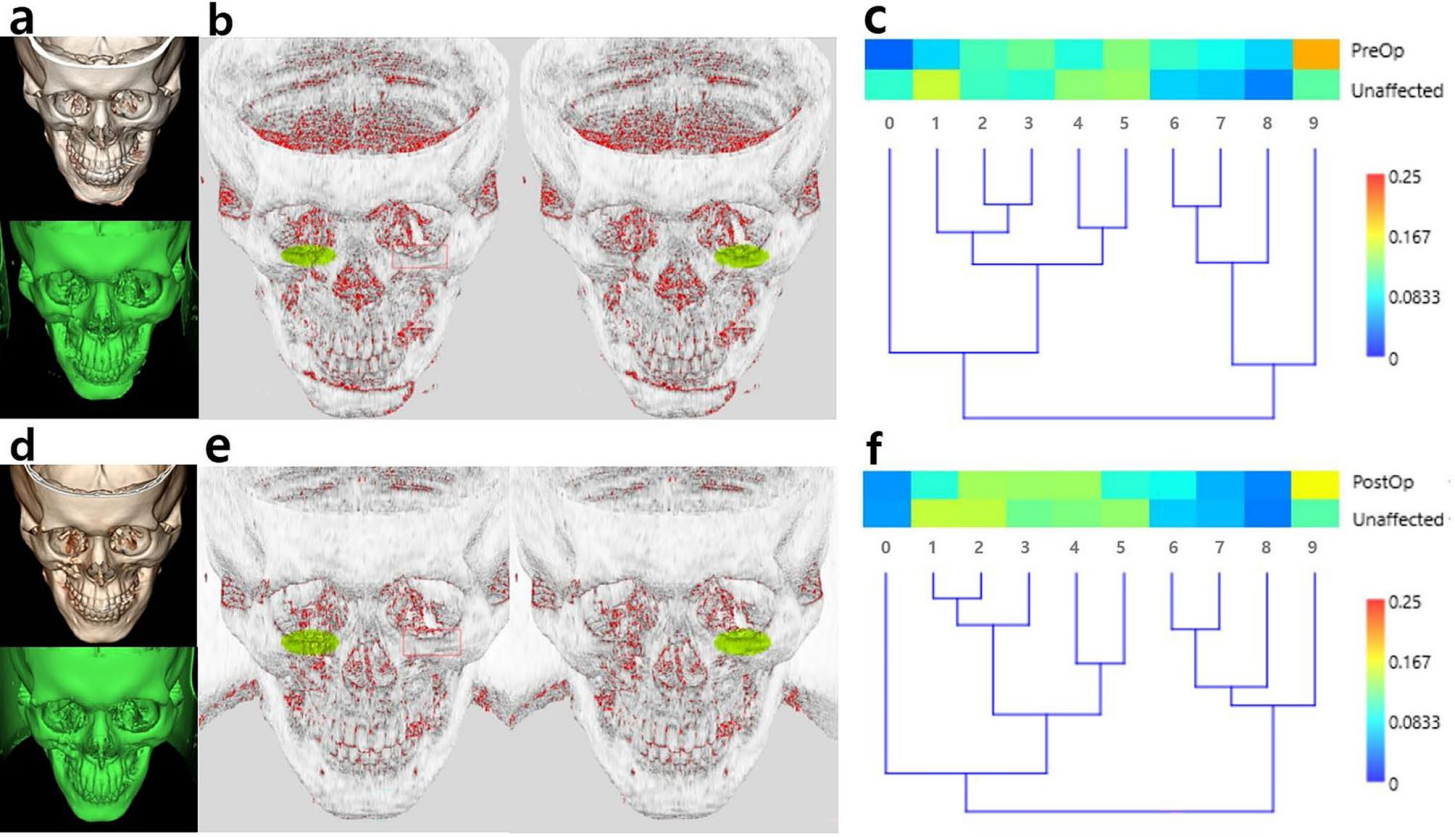
Similarity analysis of the inferior orbital rim using BCSI and hierarchical clustering. (**a**) Fractured right inferior orbital rim was presented in DICOM and OBJ images. (**b**) The subject areas on affected and unaffected sides were selected and displayed in fluorescent green. (**c**) The BCSI was 0.81, and the discrepancy between both sides showed notable components using the red–yellow–green–blue color spectrum chart. (**d**) The fracture site was reduced postoperatively. (**e**) The subject areas were selected in postoperative images. (**f**) The postoperative BCSI was 0.9 showing increment compared to the preoperative value, 0.81. The differences were improved in comparison with the preoperative analysis.

**Figure 4:**
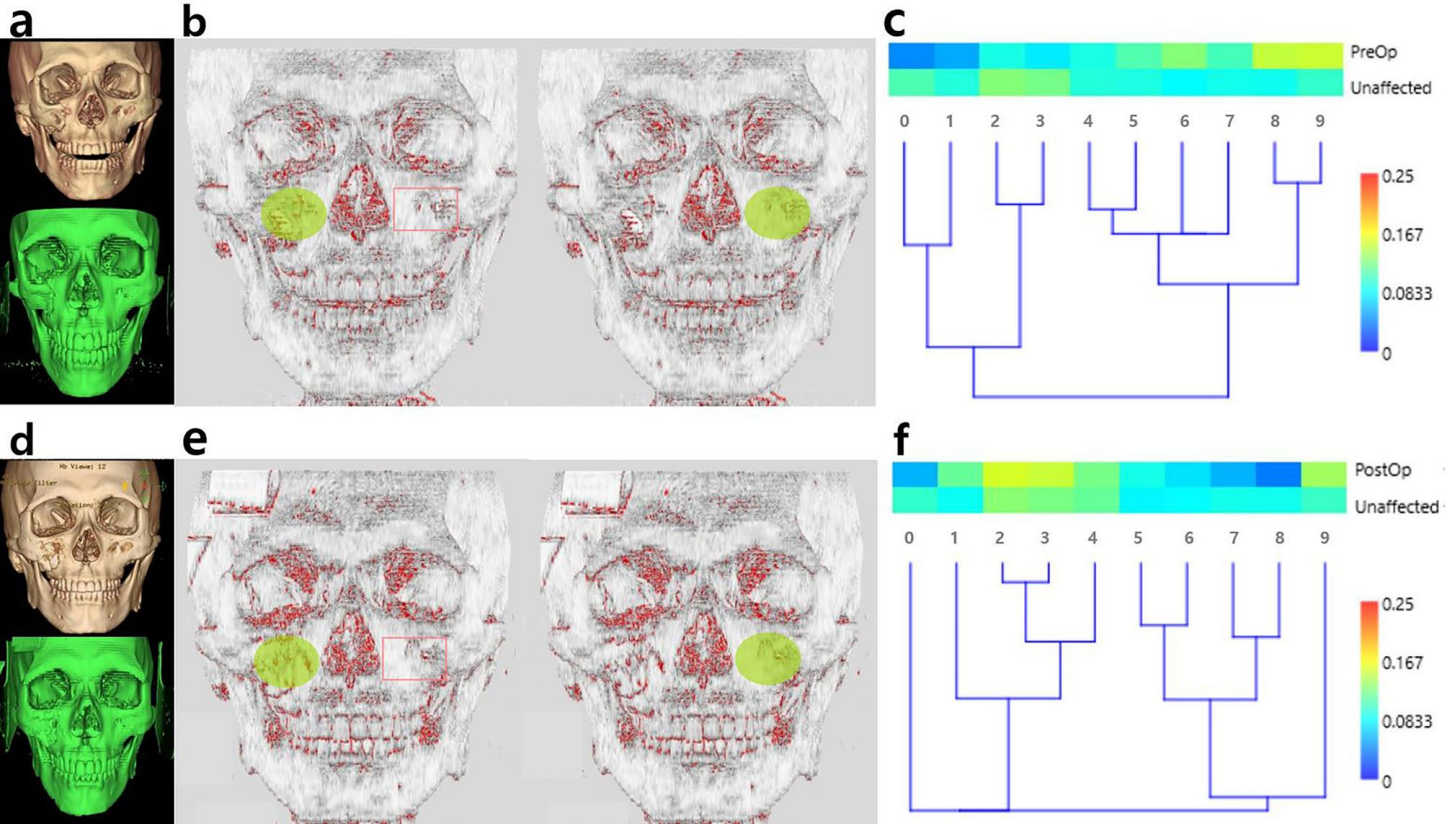
Similarity analysis of the anterior maxilla using BCSI and hierarchical clustering. (**a**) Fractured right anterior maxilla was presented in DICOM and OBJ images. (**b**) The subject areas on affected and unaffected sides were displayed in fluorescent green. (**c**) The BCSI was 0.81, and the discrepancy between both sides showed notable components using the color spectrum chart. (**d**) The fracture site was reduced postoperatively. (**e**) The subject areas were selected in postoperative images. (**f**) The postoperative BCSI was 0.87 showing increment compared to the preoperative value, 0.81. The differences were improved in comparison with the preoperative analysis.

The BCSI of the inferior orbital rim was 0.802 ± 0.041 preoperatively, and the index value was 0.904 ± 0.015 postoperatively (*P* < 0.05). Regarding the anterior maxilla, the BCSI was 0.797 ± 0.029 preoperatively, and the index value was 0.84 ± 0.025 postoperatively (*P* < 0.05). These data from two different anatomical areas (i.e., the inferior orbital rim and anterior maxilla) presented significant alterations, showing improved similarity after reduction surgery of facial bone fractures. Furthermore, hierarchical cluster analysis exhibited effective differentiation between pre- and postoperative similarities (Figs. 3 and 4). The Euclidean distance has been utilized to analyze the dissimilarity in cluster analyses of biomedical data^14^. We calculated the Z-score-normalized Euclidean distance. The average Euclidean distance of the inferior orbital rim was 3.703 ± 0.482 preoperatively, and the distance value was 1.803 ± 0.348 postoperatively (*P* < 0.05). The distance between the two datasets (i.e., data of affected and unaffected sides) decreased significantly, showing improved similarity after surgery. The average Euclidean distance of the anterior maxilla was 4.792 ± 0.559 preoperatively, and the distance value was 3.272 ± 0.813 postoperatively (*P* < 0.05). The distance decreased significantly on the anterior maxilla, showing improved similarity. The raw dataset is presented as a supplementary file (supplement 1).

The intra-observer reliability was evaluated in five random facial bone CT images. The reliability of curvature measurements on the inferior orbital rim ranged from 0.87 to 0.92. The reliability of measurements on the anterior maxilla ranged from 0.83 to 0.89. The outcomes of the two regions of interest (i.e. inferior orbital rim and anterior maxilla) showed almost perfect reliability.

## Discussion

Facial bone is composed of a collection of thin segments and contains hollow structures (i.e., foramens and sinuses), which distinguishes it from long bones. Because of this difference, analysis software is an essential component for facial bone studies and image assessment^4,15^. Therefore, clinical physicians familiar with facial bone images and computer scientists who can develop image analysis software conducted a joint study.

Computational biology is a research field that focuses on data analysis and validation of theoretical methods in biological interactions. Adequate analytic software is crucial because non-invasive evaluation and preliminary studies are required. This type of analysis is particularly important in biomechanical sciences^16^. Many studies have been performed using analytic software to detect and estimate subtle changes. Ylitalo et al.^17^ quantified the surface of degenerated cartilage using micro-CT image stacks. Surface continuity, fibrillation, and fissures were examined to evaluate osteoarthritis on articular surfaces. This method could provide an additional type of reference methodology for diagnostic imaging. In addition, Salfer et al. estimated the curvature of cell membranes using cryo-electron tomography images^18^. An estimation algorithm was introduced to differentiate reliable signals in noisy image data. The analytic strategy was also applied to the surface of embryonic cells and human brain hemispheres.

Recently, Kuchel et al.^19^ presented a strategic measurement of a single red blood cell (RBC) that moves dynamically. The surface of RBC shows both concave and convex characteristics. Mesh-triangle areas have been expanded and diminished to reach a more accurate outcome. The geometry of the total curvature was verified based on the Gauss-Bonnet theorem. Chang-Gonzalez et al.^20^ constructed a three-dimensional model to analyze the morphology of early-stage zebrafish embryo. The living creature is too tiny to visualize, so simple geometric figures, such as beads and bonds were utilized to reproduce the appearance. Time-series microscopy images were acquired and they were stratified for detailed estimation. The latest studies can be utilized to analyze subtle changes in CT images before and after surgery. Furthermore, the skillful method can lead to data correction for more accurate images, since patients may move a little bit during image acquisition.

Our analytic software emphasized the need for an adequate algorithm to evaluate the degree of curvature that can provide straightforward visualization and data expression in understandable ways. A geometric algorithm was suggested based on the analytic strategy of clinicians. Accordingly, reasonable calculation formulas that could be understood by scientists from different research fields were necessary. Therefore, a triangular mesh model was adopted to analyze bent and curved surfaces. The initial medical images were stored in a DICOM file format and converted into OBJ files, which are versatile and can be utilized for 3D image assessment^21^. The OBJ image files contain multiple triangles sharing vertices and sides. These elements provide vectors and a reference plane. The average sin values of the angles between each vector and the reference plane were utilized to produce a discrete curvature approximation.

During the program development process, the range of the triangular mesh (triangular pyramid) that should be used to calculate the curvature was discussed. When the curvature is calculated by specifying a wide range based on one apex, irregularities can be more sensitively measured. Therefore, even small changes can result in a large value (i.e., close to 1). During the initial program development, the results were derived from a narrow range setting, and thus the difference between the fractured part and the normal part was not evident. To solve this problem, the range was gradually increased, and a range over which the results were valid was obtained.

The analytic software has user-friendly features in the image analysis stage. OBJ files are input into the software and evaluated by clinicians. The subdivisions among facial bone structures can be easily selected using a mouse cursor. Thereafter, a bar graph showing curvature ranges and the percentage of each curvature range is presented automatically. Numeric values (i.e., discrete curvature approximation) are also displayed in a different window. This simplicity added efficiency and accessibility to the analysis process. Furthermore, the practicability of our software can be emphasized in serial analysis of long-term follow-up patients. Multiple postoperative CT images are available when the patients undergo assessments periodically. Our software demonstrates discrete curvature values of fractured site, and can be applied at immediate postoperative stage as well as long-term follow-up when adequate bone union has been achieved. The curvature analysis results possibly provide objective information of the area for further researches.

Before surgery, both the inferior orbital rim and the anterior maxilla exhibited greater curvature values due to sharp bends. After surgery, a decrease in the curvature value occurred because of substantial changes after reduction (Figs. 1 and 2). In addition, when the fractured side and the unaffected side were compared with each other, greater similarity results were demonstrated after surgery using the BCSI (Figs. 3 and 4). In the similarity analysis, the curvature values of unaffected sides were measured in CT images before and after surgery. The CT images at different stages could demonstrate a discrepancy of unaffected areas. We discussed the equalization of data, however such modification could result in unexpected errors. Therefore original data were utilized, although the curvature values were not identical.

Because of their anatomical nature, neither the inferior orbital rim nor the anterior maxilla forms a perfect curve^22,23^. Therefore, an indicator is needed that can be used as a standard for analysis. Therefore, to increase the objectivity of measurements, comparisons were performed before and after surgery, followed by comparisons between the fractured side and the unaffected side. Facial bone curvature has been studied due to its complexity and regional shape changes during the aging process. Williams et al.^24^ performed a thorough analysis of the curvature of several landmarks. Data were collected on the orbit, zygomatic arch, nasal bone aperture, and alveolar process of the maxilla. The configuration and vector plot of each structure provided useful information to perceive the curvature of facial bone.

The objective measurement of curvature has been utilized in the preoperative planning stage of various studies. Facciuto et al.^25^ generated a 3D skull model of a patient who underwent composite reconstruction of the frontal and superior orbital rim areas. A xenohybrid bone substitute was used to supplement the defect, and a digital design of the curved surface was used to perform adequate reconstruction of the complicated architecture. Wang et al.^26^ designed a digital template to restore the orbital rim, orbital floor, and anterior maxilla of facial cleft patients. The outer table of the cranial bone was used as a donor site, and accurate measurement of the shape and curvature was crucial. A computer-assisted rapid prototyping technique assisted in performing precise surgical procedures and anticipating the contour of recipient sites. Park et al.^27^ presented the anatomical characteristics of patients with orbital wall fracture. Orbital CT images were analyzed according to the area and thickness of the orbital wall and the coefficient of curvature. The coefficient of curvature was measured on the inferior orbital wall (i.e., orbital floor), and the inferior wall fracture group had a greater curvature coefficient than the medial orbital fracture group.

The limitations of our study include discrepancies in outcomes depending on the image quality. The facial bone CT images were obtained using a 2 mm interval between consecutive sections. Reconstructed 3D CT images based on section slices cannot show a perfect curve, and the unintended flaws could affect the resulting values. Recent studies have presented curvature estimation and segmentation techniques to revise the breaks in curves, and favorable results have been demonstrated^2,15,18^. Our group is planning to implement appropriate strategies to resolve this limitation. Furthermore, absorbable fixation plates and screws were visible on CT images, and these could be inevitable artifacts. Advanced methods to differentiate implants from natural bony components are necessary to achieve an accurate analysis. Another limitation is that the current software has not been compared to other strategies for verification. We focused on the validation of adequate differentiation between pre- and postoperative curvature values, and recovery after fracture reduction surgery. Nonetheless, Chun et al. reported that the proposed method led to comparable outcomes with regard to Taubin’s algorithm in polyhedral curvature approximation^28,29^. Clinical application of newly developed software gives a feasibility, and various analyzing methods can provide further opportunity to evaluate in their own setting.

Appropriate analytic software is an essential component for preoperative planning and postoperative analysis. This method has the advantage of allowing delicate and non-invasive assessments. Our newly developed analysis software was applied to differentiate between pre- and postoperative curvature values. Its capability was further validated with regard to the similarity between facial bones on both sides of patients. CT images can be processed and evaluated using discrete differential geometry methods. Modification of previous formulas will foster further precision and accuracy in various fields of computational biology.

## Methods

### Patients and data acquisition

Three-dimensional (3D) CT images of facial bone were obtained from 40 facial bone fracture patients (32 males; aged 19–80 years and 8 females; aged 25–68 years) who underwent facial bone reduction surgery during March 2017 to February 2020. The study was performed in accordance with the ethical standards of the 1964 Declaration of Helsinki and its later amendments and written informed consents were obtained from the patients. The study was approved by the institutional review board of Konkuk University Medical Center (approval number: KUMC 2022-03-013).

Facial bone fracture was diagnosed based on patients’ symptoms and facial bone CT analysis; 3D CT of facial bones was used because it can display appropriate views of both preoperative displacement and postoperative correction. The CT images were obtained using a standard CT scanner (GoldSeal™ Optima™ CT660, General Electric Company, Boston, MA, USA) in the same data acquisition setting.

The inclusion criteria of this study were as follows: (1) unilateral zygomaticomaxillary fracture; (2) bony displacement of the inferior orbital rim and maxilla; and (3) the amount of displacement was larger than 2 mm. Our study was designed to compare the fracture site preoperatively and postoperatively, and besides to analyze the discrepancy between the fracture site and the unaffected side. Open reduction surgery was performed to rectify displaced segments, and a rigid fixation procedure was then performed using an absorbable plate and screws (Osteotrans, TEIJIN Medical Technologies Co, Tokyo, Japan). In our preliminary study, the thickness of the absorbable plate was a bias factor because it was visible on CT scans. Therefore, we analyzed CT images according to prominent displacement and depression.

An analytic program was developed to compare the facial 3D CT images before and after surgery. This program was designed based on the discrete curvature of a triangular mesh model, which is a value that represents the curve angle of the surface formed with an adjacent vertex for each vertex constituting the polyhedral surface of the 3D model. This is a typical method widely used to express the characteristics of a polyhedral surface^8–10^. The initial 3D CT images were demonstrated in a DICOM file format, and switched into OBJ files using a standard software (InVesalius, Renato Archer Information Technology Center, Campinas, Brazil). The OBJ files were utilized in our image analysis procedure. Default threshold voxel intensities between 226 and 3021 Hounsfield units (HU) were selected for the OBJ images, and there was no additional image smoothing. Our program conducted data processing based on the following algorithm to calculate discrete curvature values.

### Geometric algorithm of the analysis software

**Figure 5:**
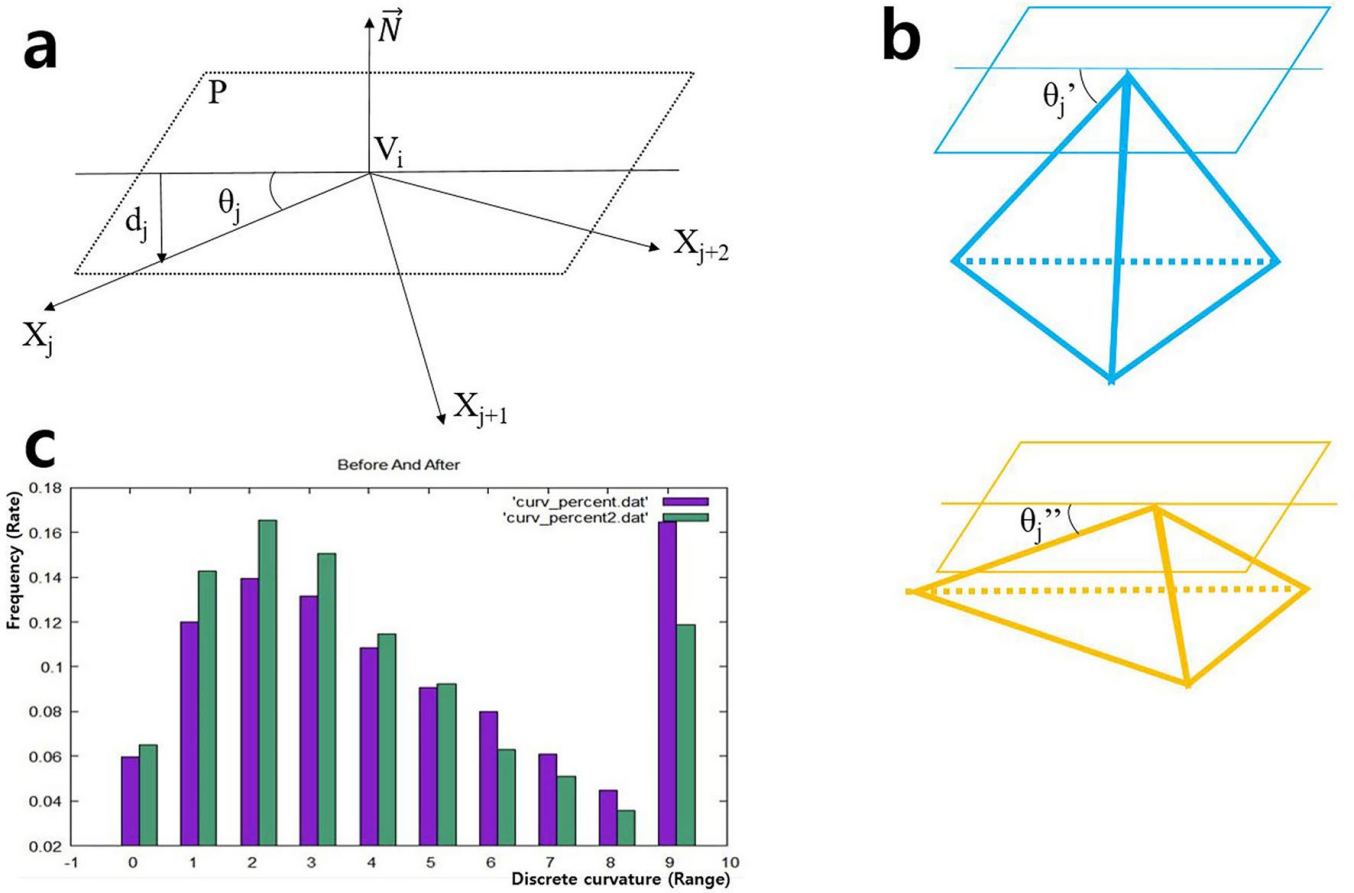
Geometric algorithm and analysis process. (**a**) Relationship between the normal vector $$\overrightarrow{N}$$ passing through V_i_ and adjacent vertices, X_j_, X_j+1_, and X_j+2_. (**b**) In calculation process, the pyramid with steep apex (blue) demonstrates larger $$\mathrm{sin}{\theta }_{j}$$ value compared to the pyramid with blunt apex (yellow). As a result, $$\sin \theta_{j}^{\prime } > \sin \theta_{j}^{\prime \prime }$$$$.$$ (**c**) The distribution of vertices according to curvature is shown before and after surgery using the Gnuplot Library. The purple bars indicate the preoperative data, and the green bars indicate the postoperative data.

A plane passing through a reference vertex (V_i_) and a normal vector in relation to the adjacent vertices were defined. The reference vertex (V_i_) indicated each point of contact that composed a surface of OBJ image. The normal vector ($\overrightarrow{N})$ consisted of the average of three vectors, namely $\overrightarrow{{X}_{j}}, \overrightarrow{{X}_{j+1}},\mathrm{ and }\overrightarrow{{X}_{j+2}}$, and $\overrightarrow{N}$ was perpendicular to plane P. The average of the sin values, which represents the ratio of the length of the side (d_j_) to the length of the hypotenuse, was defined as the discrete curvature approximation value of the reference vertex (V_i_) (Fig. 5A).

The procedure used to calculate the suggested discrete curvature approximation value was as follows. First, the normal vectors of the tangent planes were determined for each vertex. On each vertex, the reference vertex was defined as V_i_ = (V_x_, V_y_, V_z_), and the normal vector was defined as $\overrightarrow{N}$ = (N_x_, N_y_, N_z_).

$\overrightarrow{N}$ is a normal vector for the plane P passing through the vertex V_i_. Figure 5 shows the relationship between the defined plane, reference vertex (V_i_), and adjacent vertices (X_j_, X_j+1_, and X_j+2_).

The distance between plane P and the adjacent vertex X_j_ was defined as d_j_ and was calculated using Eq. (2). The angle θ_j_, which is formed between the vertex X_j_ and the plane P, and the absolute value of the sin of the angle θ_j_ were calculated using Eq. (3).

2 $$d_{j} = X_{j} \cdot \vec{N} - V_{i} \cdot \vec{N}$$

3 $$\sin \theta_{j} = \frac{{\left\| {d_{j} } \right\|}}{{\left\| {X_{j} - V_{i } } \right\|}}$$

Assuming that adjacent vertices are evenly distributed, the discrete curvature approximation value C(V_i_) for the vertex V_i_ was obtained using Eq. (4) based on the values of Eq. (3).

4 $${\text{C}}\left( {V_{i} } \right) = \frac{1}{m} \sum\nolimits_{j = 1\sim m} {\sin \theta_{j} }$$

here m indicates the number of adjacent vertices of the reference vertex V_i_, and θ_j_ indicates the angle between the adjacent vertex X_i_ and the plane P found using Eq. (2). C(V_i_) is the approximation of curvature according to its difference compared with neighboring points (Fig. 5b). Therefore, the absolute value of sin θ_j_ was used.

### Facial bone CT analysis using the software

Indices that indicate surgical outcomes can provide an improved understanding for both patients and researchers. Fractured surfaces are assumed to be present preoperatively, and therefore the curvature will be greater than that after surgery. To clearly demonstrate the facial structures, 3D images obtained before and after surgery were displayed using a histogram format. If the average curvature is presented automatically, the histogram can act as an objective indicator.

The curvature for all vertices exhibits a value between 0.0 and 1.0. As the degree of bending becomes more severe, the curvature approaches 1.0, and smooth curves have a curvature close to 0.0. Assuming that a fractured surface is present before surgery, the curvature of the vertices would be large on average. To objectively analyze and numerically confirm the results, the range between 0.0 and 1.0 was divided into 10 equal sections, and the distribution of curvature before and after surgery is demonstrated (Fig. 5c).

In the graph, the X-coordinate shows the curvature from 0.0 to 1.0 divided into 10 units (0.1 each), which represents the discrete curvature approximation value, and the Y-coordinate shows the degree of the distribution. The graph visually confirms that the values after surgery (green bars) in the selected area have a greater distribution among lower values than those calculated before surgery (purple bars). The data distribution shifted toward the lower values after surgery.

Using the software, facial 3D CT images of the inferior orbital rim and anterior maxilla of patients were compared before and after surgery. Areas of the inferior orbital rim and maxilla were designated, and curvature values from 0.0 to 1.0 were obtained. The median values were used for each curvature section: the median value for 0.0–0.1 was 0.05, that for 0.1–0.2 was 0.15, and that for 0.9–1.0 was 0.95. The median values were multiplied by the ratio of each discrete curvature section and then summed. The summed values were compared before and after surgery.

The analysis between affected and unaffected sides was performed in a similar way. The region of interest was designated on the fracture site, and additional region was set on the unaffected side. The amount of area on both sides were the same in an automatic process, and the regions have appeared as green circles. The procedures were carried out in both inferior orbital rim (Fig. 3b and e) and anterior maxilla (Fig. 4b and e). If the region of interest did not appear exactly on the corresponding area of unaffected side, we could move the green circle to the right location. The reference points of craniofacial anthropometry were utilized to set the boundary of circle.

### Statistical analysis

A paired t-test was used to compare pre- and postoperative discrete curvature values. The Bray–Curtis similarity index (BCSI) was adopted to compare the fractured and unaffected sides. The BCSI values of pre- and postoperative images were also analyzed using the paired t-test. A 95% confidence interval was used in the analysis.

The intra-observer reliability was tested with Fleiss’ kappa statistic, and the outcome was presented in kappa coefficient. A researcher measured a random CT image three consecutive times within 1 h. The range of coefficients can be interpreted as follows: < 0, poor; 0.01–0.20, slight; 0.21–0.40, fair; 0.41–0.60, moderate; 0.61–0.80, substantial; 0.81–1.00, almost perfect reliability.

Standard software (SPSS for Windows version 25.0; IBM Corp., Armonk, NY, USA) was used for the statistical analysis. A *P*-value less than 0.05 was considered to be statistically significant.

## Supplementary Information

Supplementary Information.

## Data Availability

All data generated or analyzed during this study are included in this article and its supplementary information file.
